# The Association Between Post-Traumatic Stress Disorder and Temporomandibular Disorders: A Systematic Review

**DOI:** 10.7759/cureus.31896

**Published:** 2022-11-26

**Authors:** May W Al-Khudhairy, Aseel Al-Mutairi, Bdoor Al Mazyad, Sumia Al Yousef, Sarah Hatab Alanazi

**Affiliations:** 1 Oral Biology, Riyadh Elm University, Riyadh, SAU; 2 Dentistry, King Saud University, Riyadh, SAU; 3 Dentistry, Jouf University, Sakaka, SAU

**Keywords:** post traumatic stress disorder (ptsd), temporomandibular joint (tmj) disorders, tmj disorders, review, dentistry, temporomandibular joint disorder, post traumatic stress disorder, orofacial pain

## Abstract

The purpose of this systematic study was to discover a connection between temporomandibular joint disorders and post-traumatic stress disorder. A systematic review of observational studies on post-traumatic stress disorder and the incidence of temporomandibular joint disorders (TMD) was conducted. Electronic searches of PubMed, the Saudi Digital Library, Science Direct, the Virtual Health Library (VHL), Scopus, Web of Science, Sage, EBSCO Information Services, and Ovid were performed. There was a consensus among the reviewing examiners. Only studies with the following Medical Subject Headings (MeSH) terms were included: "Posttraumatic stress disorder" combined with "temporomandibular joint disorder," "myofascial pain," "orofacial pain," "internal derangement," "disc displacement with reduction," or "disc displacement without reduction." Only full-text studies in the English language published between 2010 and June 2020 were considered. Of a total of 381 articles meeting the initial screening criteria, only eight were included in the qualitative analysis. Overall, pain is exacerbated in patients with PTSD; that is, their TMD is heightened in all aspects of pain, chronicity, decreased response to conventional therapies, and the need for more potent treatment options as compared with patients with just TMD. The evidence, albeit weak, obtained from the studies included in this review suggests a relationship between PTSD and TMDs.

## Introduction and background

The term "temporomandibular disorders" (TMDs) refers to a collection of musculoskeletal illnesses that affect the temporomandibular region. These diseases are characterised by discomfort in the temporomandibular joint, the masticatory muscles, or both [1]. TMDs were traditionally defined by the presence of at least one symptom; this has undoubtedly resulted in a wide range of observations, as some signs and symptoms, such as TMJ sound and jaw tiredness, occur in a milder and more common form. In addition, facial pain and limited mouth opening are presented in a more serious and often less widely mentioned form [2]. TMD has been considered a subclassification of musculoskeletal disorders and is a major cause of non-dental pain in the orofacial region [3].

Individuals who are stressed are clearly at a higher risk of TMDs [4]. There is a known link between TMD and stress; the development of TMD is heavily influenced by psychological risk factors such as depression, anxiety, and stress [5,6]. A considerable number of studies have proven that stress is an influential factor in the prevalence of TMDs. A study concluded that stress is highly linked to the increase in the incidence of TMDs, and patients with chronic TMDs were found to have elevated rates of anxiety [7]. Additionally, a Manfredini study discovered that people with orofacial pain had higher levels of anxiety [8]. The following comorbid conditions associated with TMDs include and are not limited to fibromyalgia [9], headaches or migraines [10] symptoms of depression and anxiety [11], and post-traumatic stress disorder (PTSD) [12, 13]. PTSD was discovered to be the second most prevalent psychological disorder, after depression, among patients with orofacial discomfort. [14].

According to the American Psychiatric Association, PTSD is defined as "the development of characteristic symptoms following exposure to an extreme traumatic stressor involving the direct personal experience of an event that involves actual or threatened death, serious injury, or other threat to one’s or another's physical integrity" [15]. War is characterised as a major stressor; it is a situation that causes psychological tension of varying degrees that could give rise to PTSD [16]. TMD and war trauma have been linked in studies on Croatian war veterans. Combat veterans with PTSD had more symptoms of orofacial pain, headache, clicking, and crepitus of the temporomandibular joint [17, 18]. Earlier studies have indicated that someone with TMD is more likely to have undergone a traumatic life event than someone without TMD [19]. At least 49.7% of chronic TMD patients had at least one traumatic life experience [20].

Multiple authors agreed that chronic pain can frequently co-occur with PTSD. A study conducted to examine orofacial pain symptoms in war veterans found that these symptoms are more widespread in individuals with PTSD [21]. An astounding 80% of these veterans reported chronic pain conditions. Studies show that the oral health status of patients with PTSD is exceedingly affected compared to control subjects, as almost half of the PTSD group were diagnosed with myofascial pain and orofacial pain [22, 23]. While pain may be related to physical illness, it is essential to remember that psychological factors have an undeniable effect on pain [24]. Several studies have found that orofacial pain, when combined with PTSD, increases pain intensity and duration while decreasing the pain threshold [20].

The co-occurrence of TMD and PTSD in an individual can further complicate the treatment of either condition [25]. It has been shown to exacerbate the symptoms of TMD in war veterans [17]. Some authors recommend that patients who experience chronic pain should be evaluated for PTSD since it is a common side effect of TMD [25,26]. It has also been proposed that the management of PTSD could be the key to obtaining pain relief in individuals who suffer from both PTSD and chronic pain [20, 27].

## Review

The Preferred Reporting Items for Systematic Review and Meta-Analysis (PRISMA) standards were followed in the reporting of this systematic review [28]. An institutional review board's approval was not required because of the nature of the current investigation. This review was submitted to the International Prospective Register for Systematic Reviews with the registration code CRD42020191809.

Eligibility criteria

The PECO approach (Population, Intervention, Incidence, Comparison, Outcomes) was used to develop the focused question in this study, where P is the population with TMD, E is the population with PTSD, C is the population without PTSD, and O is the association between PTSD and TMD (the odds of getting TMD if the person has PTSD) [29]. The inclusion criteria were observational studies addressing the relationship between TMDs and PTSD that were written in English and published between 2010 and 2020. In order to assess the levels of evidence (LoE), only publications with levels I through III were considered [30-48]. The ensuing exclusion standards were used: studies with no control group, reviews, letters, conference abstracts, personal opinions, case reports, and laboratory research; studies in which TMD assessment methods (self-report, clinical examination) were not reported or sufficiently described; studies in which there was no control group; and studies in which the full text was not available or was not available in English.

Sources of information and search strategy

Electronic searches of PubMed, the Saudi Digital Library, Science Direct, the Virtual Health Library (VHL), Scopus, Web of Science, Sage, EBSCO Information Services, and Ovid were performed. The following keywords were used to identify relevant literature: "post-traumatic stress disorder" combined with "temporomandibular joint disorder", "myofascial pain", "orofacial pain", "internal derangement", "disc displacement with reduction", or "disc displacement without reduction." A reference manager programme called Endnote X9 was used to compile references and weed out duplicates.

Study selection

All found references' titles and abstracts were checked. Studies that did not meet the eligibility requirements were disqualified. Following the first exclusion, eligibility criteria were applied to the complete texts of the studies. The decision to select a study was made by consensus.

**Figure 1:**
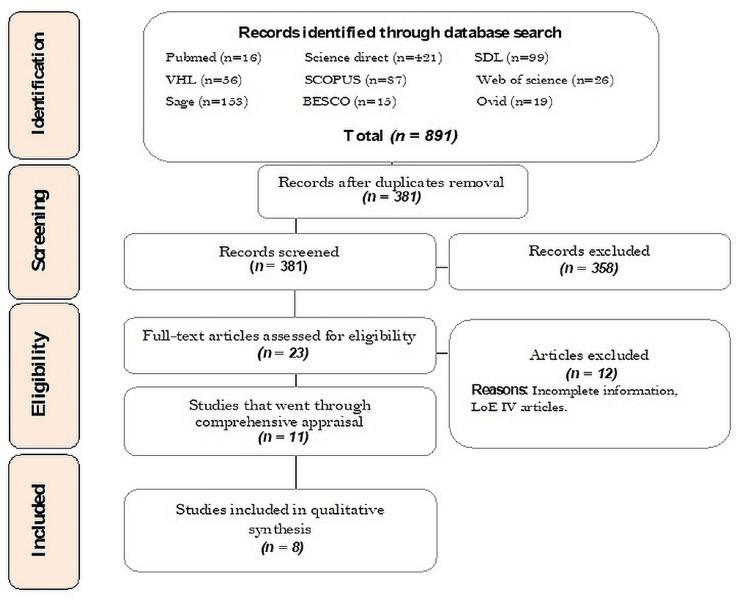
Flowchart to showcase the article inclusion process

The data collection process and data items are shown in Figure 1.

Half of the studies had material that two reviewers could use. The other two reviewers obtained the necessary information from the second half of the research. The reviewers then switched to other research and extracted the necessary data to verify the accuracy of the data gathered. Any disagreements were talked through and resolved with the fifth reviewer, ensuring inter-examiner dependability in the process.

The information gathered covered study details (authors, year of publication, country, and study type), population characteristics (sample size, gender), the methodology used to evaluate TMD and PTSD, and key conclusions, as shown in Table 1.

**Table 1:** Studies Included in the Qualitative Synthesis. TMD:  temporomandibular disorder; PTSD: post-traumatic stress disorder; MSD: musculoskeletal disorders; ICD: International Classification of Diseases; F: female; M: male; DSM-IV: Diagnostic and Statistical Manual of Mental Disorders; RDC/TMD: Research Diagnostic Criteria for Temporomandibular Disorders; DC/TMD: diagnostic criteria for temporomandibular disorders; OHIP-14: Oral Health Impact Profile-14

Authors (Year)	Study design	Country	Sample (gender)	Methods of diagnosis	The Results
Lopes et al (2022) [6]	Case-Control	Brazil	197 participants (105 with TMD and 92 controls)	Participants' responses to electronic questionnaires were evaluated before and after the first lockdown caused by the COVID-19 epidemic. The TMD Pain Screener, Numeric Rating Scale, Patient Health Questionnaire–4, and PTSD Checklist–Civilian Version questionnaires were used to evaluate the existence of potential TMD, painful severity, distress, and PTSD symptoms.	Compared to the control group, individuals suffering from TMD displayed higher degrees of discomfort both during and following the lockdown. Even though the amount of pain was the same during both evaluation periods, the TMD group also reported higher distress levels in the post-lockdown period than in the lockdown phase. Additionally, participants with TMD had a 3.91 times higher likelihood of being female and a 3.82 times higher likelihood of exhibiting PTSD symptoms following the lockdown.
Fenton et al. (2018) [38]	Cohort	USA	4.1 million veterans had MSDs; 12,626 had TMD (0.31%). (2,844 females with TMD, 250,519 females without TMD) (9782 males with TMD, 3,864,843 males without TMD)	ICD codes extracted from the Veterans’ Health Administration records were: "Risk of recall for bias error", "misclassification", and "misdiagnosis."	0.31% of the overall sample had TMDs. In women, PTSD was not significantly related to TMD; in contrast, in men, it was significantly related to TMD. Of the 2,844 women with TMD, 12.8 % had PTSD. Of the 9782 men with TMD, 13.6% of them had PTSD.
Fillingim et al. (2011) [43]	Case-Control	USA	1,633 TMD-free controls and 185 TMD cases	For this study, controls and cases completed a battery of psychosocial instruments. Factors like general psychosocial adjustment and personality, affective distress, psychosocial stress, somatic awareness, pain coping, and catastrophizing were assessed.	All of the Symptom Checklist 90-Revised (SCL-90R) subscales of primary concern in this analysis—depression, somatization, anger, and anxiety—had higher mean scores in TMD cases than controls. The average scale scores in cases were nearly twice as high as the average for controls. On the State-Trait Anxiety Inventory (STAI), TMD subjects reported greater mean levels of state and trait anxiety compared to controls, and on the Perceived Stress Scale (PSS), TMD cases showed higher mean levels of perceived stress. TMD sufferers also showed greater mean levels of catastrophizing than controls on the Pain Catastrophizing Scale (PCS).
Solis et al. (2017) [36]	Case-Control	Brazil	76 subjects: 38 PTSD patients (F: 32; M: 6). 38 controls (F: 30, M: 8)	PTSD: Structured Clinical Interview for DSM-IV TMD: RDC/TMD	The findings indicated a significant association of PTSD with some degree of orofacial pain. The PTSD group differed significantly in chronic pain grade classification, jaw functions, grinding habits, TMJ sounds, pain on muscle palpation, and pain during jaw movements. Also, they exhibited a lower RDC/TMD axis II profile.
Muhvićurek et al. (2015) [18]	Case-Control	Croatia	152 male subjects: 52 veterans with PTSD; 50 veterans without PTSD; 50 healthy men	PTSD: Structured Clinical Interview for DSM-IV TMD: RDC/TMD Gender Discrimination	There was a statistically significant difference in pain perception in PTSD patients compared to other groups. PTSD patients mostly evaluated pain on palpation as moderate or severe and had statistically significantly more painful sites.
Afari et al. (2008) [17]	Cohort	USA	630 monozygotic and 239 dizygotic female twin pairs	The Impact of Events Scale (IES), with scores divided into terciles, was used to evaluate PTSD symptoms. An inquiry about ongoing or recurrent pain in the jaw, temple, area in front of the ear, or ear for the previous three months was used to assess TMD pain. The association between the IES and TMD pain was investigated using random effects regression models that were controlled for demographic characteristics, depression, and familial/genetic variables.	TMD discomfort was strongly correlated with IES scores. Even after adjusting for demographic variables and depression, twins in the highest IES tercile were over three times more likely than those in the lowest tercile to report TMD pain, showing a substantial correlation between PTSD symptoms and TMD pain.
Tay et al (2019) [13]	Cross-sectional	Singapore	2043 participants (1998 men, 45 women; mean age: 24.18 ± 7.18 years)	The study was conducted across 12 military dental centres using a self-administered questionnaire comprising demographical data, a DC/TMD symptom questionnaire, the OHIP-14, and the Depression Anxiety and Stress Scale 21 (DASS-21).	TMD symptoms of various types and levels had varying effects on OHRQoL and psychological states. There were small but significant correlations between the number of TMD symptoms and quality of life, sadness, anxiety, and stress.
Knibbe et al. (2022) [18]	Cross-sectional	The Netherlands	673 participants were referred by their general practitioner, psychologist, or psychiatrist to the Dutch psychotrauma expertise centre for the treatment of severe PTSD.	Patients were evaluated for painful TMD (TMD pain screener), awake bruxism (AB) and sleep bruxism (SB) (Oral Behaviours Checklist), PTSD symptoms (Clinician- Administered PTSD Scale), and type of traumatic events (Life Events Checklist) prior to treatment.	Painful TMD, AB, and SB were more prevalent among patients with PTSD (28.4%, 48.3%, and 40.1%, respectively) than in the general population (8.0%, 31.0%, and 15.3%).

Risk of bias in individual studies

The risk of bias in the included studies was evaluated using the Risk of Bias Assessment Tool for Non-Randomized Studies (RoBANS), which is similar to the Cochrane Risk of Bias tool [31]. This is depicted in Table 2.

**Table 2:** Risk of bias assessment tool for non-randomized studies (RoBANS) LoE: levels of evidence

	Selection of participants	Confounding variables	Blinding of outcome assessment	Incomplete outcome data	Selective outcome reporting	LoE
Lopes et al (2022) [6]	Low	Low	Unclear	Low	Low	I
Fenton et al. (2018) [38]	Low	Low	Low	Low	Low	I
Fillingim et al. (2011) [43]	High	High	Unclear	Low	Low	I
Solis et al. (2017) [36]	Low	Unclear	Unclear	Low	Low	III
Muhvićurek et al. (2015) [18]	Low	Low	Unclear	Low	Low	III
Uhac I et al. (2014) [17]	Unclear	Unclear	Unclear	Low	Low	III
Tay et al (2019) [13]	Low	Low	Unclear	Low	Low	
Knibbe et al. (2022) [18]	Low	Unclear	High	Low	Low	

Inter-examiner reliability was ensured by dividing the articles between the reviewers and switching them around. The fifth reviewer mediated in situations of disagreement.

Synthesis of results

We summarised the data using a descriptive approach (qualitative analysis) and performed a quantitative analysis (meta-analysis) of the extracted data from the case-control studies.

Quality assessment

Studies that were included in this systematic review received quality ratings using the Newcastle-Ottawa Scale, which increased the strength of this study (NOS) [32, 33]. This is shown in Table 3.

**Table 3:** Quality assessment criteria used for cohort studies through the Newcastle‐Ottawa Scale

	Lopez et al., (2019)	Fenton et al. (2018)	Fillingim et al. (2016)
Selection: Representativeness of the exposed cohort: truly representative of the average in the target population. (random sample or whole population); somewhat representative of the average in the target population (non‐random sampling); a selected group of users e.g., nurses, volunteers; no description of the derivation of the cohort	a*	a*	b*?
Selection of the non-exposed cohort: drawn from the same community as the exposed cohort; drawn from a different source; no description of the derivation of the non-exposed cohort	c	a*	c
Ascertainment of exposure: secure record (e.g., surgical records); structured interview; written self-report; no description	b*	a*	a*
Demonstration that outcome of interest was not present at the start of the study: yes or no	b	B	b
Comparability: Comparability of cohorts on the basis of the design or analysis: the study controls for the most important factor (select one): the study control for any additional factor (This criterion could be modified to indicate specific control for a second important factor)	a*?	a*?	a*?
Outcome: assessment of the outcome: independent blind assessment; Record linkage; self‐report; no description	a*	b*	a*
Was the follow-up long enough for outcomes to occur: yes (select an adequate follow-up period for the outcome of interest); no	a*	a*	a*
Adequacy of follow-up of cohorts: complete follow-up (all subjects accounted for); subjects lost to follow-up unlikely to introduce bias (small number); follow-up rate ____% (select an adequate %); and no description of those lost; no statement	b*	a*	a*
Score	6/9	7/9	6/9
	Solis et al. (2017)	Muhvić-Urek et al, (2015)	Tay et al (2019)
Selection: is the case definition adequate? Yes, with independent validation; yes, e.g., record linkage or based on self-reports; no description	a*	a*	a*
Representativeness of the cases: consecutive or obviously representative series of cases; potential for selection biases or not stated	a*	a*	b
Selection of controls: community controls; hospital controls; no description	b	a*	c
Definition of controls: no history of the disease (endpoint); no description of source	a*	a*?	b
Comparability: comparability of cases and controls on the basis of the design or analysis: the study controls for the most important factor (select one); the study control for any additional factor (this criterion could be modified to indicate specific control for a second important factor.)	a*?	a*?	a*?
Exposure: ascertainment of exposure: secure record (e.g., surgical records); structured interview where blind to case/control status; interview not blinded to case/control status; written self-report or medical record only; no description	c?	b*?	e
The same method of ascertainment for cases and controls: yes or no	a*	a*	b
Non-response rate: same rate for both groups; Non-respondents described the rate differently and no designation	a*	a*	a*
Score	6/9	8/9	3/9

Similar to how RoBANS was evaluated, a quality assessment was also conducted. For cohort studies, the NOS assigns a score based on the examination of three categories (i.e., selection, comparability, and outcome), and for case-control studies, three categories (i.e., selection, comparability, and exposure). A study may receive a maximum of one star, in accordance with NOS rules, for each numbered item within the selection and the exposure or outcome categories. For comparability, a maximum of two stars may be given. Therefore, research of the highest calibre is given a score of up to nine.

Discussion

This systematic review aimed to investigate the association between the signs and symptoms of post-traumatic stress disorder (PTSD) and temporomandibular disorders (TMDs). The DSM-IV, 1994 [34] has been the most commonly used in four of our included studies for the diagnosis of PTSD [13, 22, 35, 36]. A person must have been exposed to a traumatic incident (criterion A), have at least one re-experiencing symptom (criterion B), three avoidance symptoms (criterion C), and numerous hyper-arousal symptoms in order to meet the DSM-IV criteria for PTSD (criterion D) [34, 37].

One study published in 2019 examined the correlations between TMDs and PTSD criteria. In particular, criterion D (hyperarousal symptoms). This study, which was conducted on a representative sample of northeastern Germany's general population, is population-based. According to the findings in Table 1, there is a moderate-to-substantial correlation between PTSD symptoms and TMD. In comparison to patients without clinical PTSD, subjects with clinical PTSD showed a 2.56-fold increase in joint pain and a 3.86-fold increase in muscle discomfort. Additionally, the odds of having joint pain and masticatory muscle pain were both 3.04 and 3.37 times higher, respectively, in people with D-criterion symptoms. Additionally, they were 2.8% more likely to fall into a higher pain group than people who did not exhibit D-criteria symptoms [35]. However, this study, although included in our review, does pose a certain degree of attrition bias since a considerable number of the subjects were lost in the follow-up process.

In 2018, a cohort study involved data obtained from the musculoskeletal disorder (MSD) cohort, which included 4.1 million veterans. TMD affected 12,626 (0.13%) of the total sample. Stratification by sex revealed that 12.8% of women with TMD also had PTSD. While 13.6% of men with TMD had PTSD, they found that the odds of PTSD were not significantly related to TMD in women. In contrast, men had significantly higher odds of TMD associated with PTSD [38]. It is noteworthy to mention that this study included participants who had been selected by their ICD codes, meaning that their diagnoses were based on their health records. This, in turn, stipulates a study that may be at risk for recall bias and be subject to error, misclassification, and misdiagnosis.

The Research Diagnostic Criteria for Temporomandibular Disorders (RDC/TMD), which was developed in 1992, has been the most widely used diagnostic method for TMD [39]. It was widely used by clinicians and researchers, and its reliability and validity had been proven [40]. In this systematic review, RDC/TMD was used in four of the included studies [36,41,42]. Additionally, the Orofacial Pain Prospective Evaluation and Risk Assessment (OPPERA) cooperative agreement was used to evaluate the symptoms of PTSD [43]. The PCL-C is defined as "a self-report questionnaire that measures the number of traumatic experiences and the extent of PTSD symptoms associated with these experiences" [44,45].

A patient's pain score of 41 on the PCL-C scale is regarded as a valid and reliable cut-off score to assess if an individual has recorded symptoms consistent with a DSM-V diagnosis of PTSD [34,46]. In this retrospective study, 610 participants were included in their research to study the effect of the interaction between PTSD and smoking on pain intensity and pain-related functioning with regard to orofacial pain. The results demonstrated that 22% of the subjects were found to be diagnosed with PTSD. They concluded that, among TMD patients, the degree of PTSD symptoms strongly predicted increases in pain, psychological distress, and pain-related functioning [43]. In five of the included case-control studies [13,36,41,42,47], the PTSD diagnosis was made by a psychiatrist. As for the TMD diagnosis, three studies [36, 41, 42] utilised the RDC/TMD protocol. Data from all of these studies showed significant values when comparing PTSD groups to their controls.

According to a 2017 case-control study, there were substantial differences between the control and PTSD groups in terms of how chronic pain was graded. The RDC/TMD axis II profile score, pain levels during excursive jaw movement, and extra-oral muscular discomfort were all higher in the PTSD group [36]. Similar findings were found in a study published in 2002 [42]. There was a statistically significant difference in the way the PTSD group perceived pain compared to their controls, and the PTSD group also had considerably more painful areas than the control group. In a 2014 60-participant case-control study, there was a significant difference in the level of joint and muscle pain between subjects with and without PTSD, along with a substantial difference in the frequency of TMD symptoms in subjects with PTSD [41].

In addition, in a study on TMJ health in war veterans with PTSD [13], researchers performed a clinical assessment of multiple signs and symptoms of TMJ (despite a lack of reference for their examination technique), and their results were consistent with the aforementioned study [41]. Compared to the other groups, veterans experienced more severe discomfort when the masseter, temporal, pterygoideus, digastric, and sternocleidomastoid muscles were palpated. Additionally, there was a statistically significant difference between the groups in both the frequencies and the clicking noise made when chewing. They concluded that, compared to controls, PTSD-affected war veteran individuals had considerably worse TMJ functional status [13]. Moreover, this study poses a discrimination bias since the participants were all male veterans. Similarly, a Croatian case-control study aimed to investigate TMJ pain in war veterans with PTSD [47]. In this study, there was a detailed log of both the examination technique for TMJ and its reliability and validity [48, 49]. It was reported that the left lateral pterygoid site was the most frequent painful location in the PTSD group, and they had significantly more frequent joint sensitivity than their control group [47].

A rationale for the underlying association between PTSD and TMD may be attributed to the lowered pain threshold of PTSD sufferers caused by abnormal pain processing in the trigeminal system [50]. PTSD affects motor function, causing neurotransmitter disturbances and increasing muscle tone [17, 51]. This is especially apparent in the muscles of the head and the face in lieu of their actions as the muscles of facial expression [52]. The infamous "mutual maintenance" theory suggested by Sharp and Harvey [53] popularised the idea that PTSD symptoms are maintained and exacerbated by pain and vice versa. It is well established by a wide body of literature that an individual with PTSD may experience considerably higher levels of pain symptoms. While pain may be related to physical illness, it is essential to remember that psychological factors have an undeniable effect on pain [24]. Multiple studies have stated that orofacial pain, when accompanied by PTSD, increases pain intensity and duration and lowers one’s pain threshold [20,23].

Furthermore, comorbidities hold true. The existence of one or more conditions, such as fibromyalgia, headaches, and depression, amongst others, with TMD is not a mere coincidence but, in fact, an expected one [38]. Oddly enough, younger men, along with being single or having an unknown marital status, experienced more TMD. In addition, the comorbidities with a higher chance of TMD were migraine, tension-type headaches, irritable bowel syndrome, major depression, PTSD, and anxiety disorders. Hence, mental health plays a role and may initiate, exacerbate, or even aggravate TMD.

Limitations

The listed studies have a lot of limitations. For instance, while choosing a sample for research, a handy sample that wasn't representative of the entire population was picked. One study had some attrition bias since a significant portion of the individuals were lost during the follow-up phase [35]. Another study relied on ICD codes to identify the individuals' diagnoses from their medical records; however, this method may be prone to mistakes, misclassification, and misdiagnosis, which puts the study at risk of recall bias [38]. Moreover, the analyses of some of the included studies were not performed on both genders [13,42,47]; that is, there was gender discrimination as these studies were conducted on war veterans, the majority of whom were male. Lastly, the clinical examinations of TMDs [13] and the psychological assessment of PTSD were not adequately described [38,41,47]. Though in studies where PTSD diagnostic tools were not mentioned, psychiatrists did the PTSD assessments, similarly to all the other studies included in this systematic review.

## Conclusions

The studies chosen for this review have demonstrated clearly how PTSD and TMDs coexist in a complex manner. TMD patients typically experience PTSD, and PTSD subjects at the same time also have a higher prevalence of TMD. It is also evident that, due to having a lower tolerance for pain, TMDs tend to be a common malady affecting people diagnosed with PTSD. Physicians should consider the correlation between PTSD and TMDs that our study has demonstrated when developing strategies for detecting and treating both conditions. Before beginning any therapy plan, it is crucial to notify such patients and include the appropriate doctors, in this case, a psychiatrist, psychologist, or expert in orofacial pain.
